# Mesenchymal R-spondin 3 promotes an immunogenic tumor microenvironment with adaptive immune resistance in human gastric adenocarcinoma

**DOI:** 10.1007/s10120-026-01791-2

**Published:** 2026-08-24

**Authors:** Anne-Sophie Fischer, Alexander Arnold, Hilmar Berger, Stefanie Müllerke, Jonas Wizenty, Hans-Joachim Mollenkopf, David Horst, Frank Tacke, Christoph Treese, Michael Sigal

**Affiliations:** 1 Department of Hepatology and Gastroenterology, Charité-Universitätsmedizin Berlin, Augustenburger Platz 1, 13353 Berlin, Germany; 2 Berlin Institute of Health, Berlin, Germany; 3 Department of Molecular Biology, Max Planck Institute for Infection Biology, Berlin, Germany; 4 Department of Pathology, Charité-Universitätsmedizin Berlin, Berlin, Germany; 5 Department of Gastroenterology, Infectiology, and Rheumatology Charité-Universitätsmedizin Berlin, Berlin, Germany; 6 Cluster of Excellence ImmunoPreCept, Charité-Universitätsmedizin Berlin, Berlin, Germany

**Keywords:** *Helicobacter pylori*, Tumor microenvironment, Inflammation, PD-L1, Intestinal-type gastric cancer

## Abstract

**Background:**

The main risk factor for gastric cancer (GC) is inflammation driven by *Helicobacter pylori* (*H. pylori*) infection. Despite growing use of immunotherapies targeting the tumor inflammatory microenvironment, responses remain suboptimal, highlighting the need to define inflammatory drivers and improve patient stratification.

**Methods:**

We assessed the role of R-spondin 3 (RSPO3), a stroma-secreted Wnt potentiator, in a Caucasian tissue microarray of 277 chemotherapy-naïve GC patients by associating RSPO3 expression with tumor inflammation, tumor stage, and survival. To mechanistically explore the role of Rspo3 in immune cell infiltration, we used conditional mouse models with stromal Rspo3 overexpression or knockout and infected them with *H. pylori*. We conditionally depleted the epithelial Rspo3 receptors Lgr4 and Lgr5 in mice to assess the mode of Rspo3 signaling. Findings were validated by generating a single-cell atlas from The Cancer Genome Atlas and performing gastric cancer transcriptome analysis.

**Results:**

Tumor RSPO3 expression positively associated with mucosal T-cell infiltration and combined positive score, a phenotype specific to intestinal-type GC. In mice, stromal Rspo3 promoted T-cell infiltration with increased interferon-γ signaling and PD-L1 expression in *H. pylori*-driven precancerous lesions. Epithelial depletion of Lgr4/5 reduced gastric T-cell infiltration and PD-L1 expression.

**Conclusions:**

In mice, mesenchymal–epithelial RSPO3–LGR4/5 signaling drives inflammation and enhances immune checkpoint signaling in precancerous lesions. Positive associations between RSPO3 expression and inflammation suggest that RSPO3 signaling contributes to shaping the immune microenvironment of human intestinal-type GC.

**Supplementary Information:**

The online version contains supplementary material available at https://doi.org/10.1007/s10120-026-01791-2.

## Introduction

Gastric cancer (GC) is the third leading cause of cancer-related deaths worldwide [1], and approximately half of patients are diagnosed at advanced stages requiring systemic therapy [2]. Although immunotherapy has improved outcomes in GC, many patients fail to respond. Established biomarkers, including PD-L1 expression and lymphocyte infiltration, only partially predict therapeutic benefit, and the mechanisms underlying the heterogeneous immune landscape of GC remain incompletely understood. Identification of factors responsible for differential immunological states may not only provide new insights into the mechanisms driving gastric carcinogenesis but also allow the identification of new reliable biomarkers that could improve response prediction.

*Helicobacter pylori (H. pylori)*, the major risk factor for GC, induces chronic inflammation and premalignant epithelial changes that can progress to cancer [3]. Beyond tissue adaptations driven by chronic inflammation, *H. pylori* alters stromal signaling pathways, including the Wnt/Rspo3 axis [4], which regulates epithelial proliferation and differentiation [5–7]. R-spondin 3 (RSPO3) is a stromal ligand that potentiates Wnt signaling through the epithelial receptors LGR4 and LGR5. In mouse models, *H. pylori*-induced Rspo3 promotes hyperplasia, hyperproliferation, and metaplasia [5, 6, 8, 9]. Although aberrant Wnt signaling occurs in approximately half of human GCs [10], the contribution of RSPO3 signaling to gastric carcinogenesis remains poorly understood and may extend beyond canonical Wnt pathway activation [11].

We have previously identified *Rspo3* as a key regulator of epithelial inflammatory responses [3, 7–9], and Wnt signaling has been linked to PD-L1 expression ^12^. In gastric cancer, immunotherapy is recommended for patients with a combined positive score (CPS) ≥5, which includes the percentage of PD-L1-positive tumor cells (Tumor Proportion Score =TPS) and immune cells [13]. The CheckMate 032 study demonstrated that response rates to immunotherapy were higher when using the CPS score as opposed to an equivalent TPS score, highlighting the role of tumor-infiltrating lymphocytes for therapy response [14].

We therefore investigated whether RSPO3 expression is associated with immune cell infiltration and immune checkpoint signaling in gastric cancer.

## Methods

### Patient cohort and tissue microarray preparation

Detailed descriptions of the cohort and the tissue microarray preparation have been reported elsewhere [15–18]. In short, all available tissue samples of chemotherapy-naïve patients with gastric tumors primarily treated by surgery between 1992 and 2004 at the Charité—Universitätsmedizin Berlin that were stored in the archive of the Department of Pathology at Charité—Universitätsmedizin Berlin were collected retrospectively. In total, we retrieved formalin-fixed tumor tissue of 277 patients of all tumor stages. Per patient, two tissue samples were included in the tissue microarray. The patient cohort included 233 patients with gastric adenocarcinoma and 44 patients with adenocarcinoma of the esophagogastric junction. A detailed summary of patient characteristics is shown in Supplementary Table 1. Data on tumor stage were retrieved from the patient management software (SAP) and the regional population-based cancer registry (“Gemeinsames Krebsregister”). The mean follow-up was 101.8 months (95% CI 91.01–112.54). Survival was defined as time from diagnosis to death or last follow-up. This study was approved by the Institutional Review Board of the Charité (EA4/115/10).

### In vivo mouse experiments

All procedures involving animals were approved by the institutional and local legal authorities at the Max Planck Institute for Infection Biology as well as at Charité-Universitätsmedizin Berlin (LAGeSo, Berlin). All animals were maintained in autoclaved microisolator cages and provided with sterile drinking water and chow ad libitum. *Rspo*^*fl*/fl^ mice enable the Cre-mediated recombination and excision of exons 2 to 4 of the *Rspo3* gene [19] and were a gift from J. Cobb (University of Calgary, Calgary, Canada). *MYH11CreERT2* mice [20] were provided by S. Offermanns (Max Planck Institute for Heart and Lung Research, Bad Nauheim, Germany). To generate conditional KO mice with depletion of *Rspo3* in myofibroblasts, we bred *Rspo3*^*fl/fl*^ mice with *MYH11-CreERT2* mice, while *MYH11-CreERT2* littermates bred with WT *Rspo3* alleles served as controls. Mice that conditionally overexpress an *Rspo3* transgene under the CAGGS promoter in a *Rosa26* gene-targeting cassette (*Rosa26Sor6[Cag-Rspo3]*) were described previously [21]. These animals were bred with *MYH11-CreERT2* mice to generate double-heterozygous *MYH11-CreERT2/Rosa26Sor6(CAG–Rspo3)* mice. *Axin2-CreERT2* mice were obtained from the Jackson Laboratories [22]. *Lgr4*^fl/fl^ mice and *Lgr5*^fl/fl^ mice were kindly provided by Dr. Jan Tchorz, Novartis. Mouse models were designed for both genes by flanking exon1 with loxP elements, facilitating Cre‐mediated recombination and excision of exon1 including part of the 5′UTR. Six- to eight-week-old male mice were used for this study. Littermates were used for the experiments and randomly allocated to experimental groups. To induce KO or KI of *Rspo3* or *Lgr4/5,* tamoxifen (MilliporeSigma) was injected intraperitoneally as a single dose (4 mg/25 g body weight, diluted in 200 μl corn oil).

At the time of harvest, the forestomach was removed, and the glandular stomach was opened along the lesser curvature and laid flat. The stomach contents were removed, and the tissue was divided into 2 halves along the greater curvature. For microscopy analysis, a similar longitudinal section at the midline along the greater curvature was used in all animals to minimize sampling error. Experiments were performed in at least 3 biological replicates per condition.

### *H. pylori* infection

Mice were orally infected with a single dose of 10^8^ *H. pylori* PMSS1 and were euthanized 2 months later. A longitudinal section of stomach tissue was weighed and then mechanically homogenized in brain–heart infusion medium. Serial dilutions of the homogenates were plated for enumeration of colony-forming units, and bacterial counts were expressed as colony-forming units per gram of stomach to ensure infection efficiency. Experiments were performed in at least 3 biological replicates per condition.

### Single-molecule RNA in situ hybridization

Tissue sections cut at 5 μm thickness were processed for RNA in situ detection using an RNAscope Red Detection Kit according to the manufacturer’s instructions (Advanced Cell Diagnostics). RNAscope probes against human RSPO3 (491,461, Lot 18137A) were used; positive and negative control probes were run in parallel and used according to the manufacturer’s instructions. Images were acquired using a Zeiss Observer 7.

### Murine tissue sample preparation

Murine tissue samples were fixed in 4% paraformaldehyde for 24 h and then washed twice with PBS. Paraffinization was conducted with automated tissue processing systems at the Charité Core Unit “Immunopathology for Experimental Models”. Sectioning into 5 µm thick sections was performed by laboratory technical assistants at the same Core Facility.

### Immunohistochemistry staining and immunofluorescence labeling

Immunohistochemistry was performed by the Department of Pathology at Charité CCM using automated immunohistochemistry. For CD3, CD4, CD8, and PD-L1 staining, a Leica BOND-max™ immunostainer (Leica Microsystems) using the Bond Polymer Refine (a peroxidase-based detection reagent) was used, and slides were counterstained with hematoxylin. The following antibodies were applied: CD3 (clone LN10, NCL-L-CD3-565, Novocastra®), CD4 (4B12, CD4-368-LC, Novocastra®), CD8 (C8 / 144B, M7103, DAKO®), PD-L1 (clone E1L3N, Cell Signaling Technologies, Danvers, USA).

### Microscopy and quantification

Due to the procedure of cutting and staining, some tissue microarray samples were incomplete or washed off. Those were excluded from further analysis, leading to different absolute numbers of investigated samples for the different stainings. Microscopy was performed using a Zeiss Observer 7 microscope for immunohistochemistry staining, and immunofluorescence-labeled sections were scanned with a Leica Sp8 confocal microscope. With both microscopes, tile images were generated to map the entire tissue section. Images were edited and analyzed in ZEN 3.4 (blue edition) or ImageJ1.47v software. Infection-driven alterations in WT mice are primarily restricted to the transition zone. Therefore, and because the Lgr4/5 knockout was induced in Axin2+ cells, which predominantly occur in the antrum, positive cell numbers in Lgr4/5 KO mice and Lgr4/5 WT mice were quantified in the transition zone. *Rspo*3 overexpression led to a drastic shift of the antrum/corpus ratio towards larger corpus areas with a less obvious transition zone. Hyperplasia driven by *Rspo3*-overexpression was primarily found in the corpus with less obvious alterations of antrum pathophysiology. Therefore, quantification of positive cells in Rspo3 KI mice was performed on the corpus tissue. Quantification of CD3+/CD4+/CD8+/PD-L1+ cells was conducted semiautomatically using QuPath software, version 0.2.3.

The classification of human tissue samples into RSPO3 high/low was performed by an expert blinded to the patients’ clinical information. In detail, because the granular, dot-like staining pattern detected by RNAscope does not allow reliable continuous quantification at the single-cell level, RSPO3 expression was assessed semi-quantitatively analogous to established diagnostic scoring systems such as HER2. Tumors with absent or faint equivocal signal were classified as RSPO3-low, whereas those with unequivocal moderate to strong signal were classified as RSPO3-high. This threshold ensures reproducible scoring above background while reflecting the biology of RSPO3, which acts as a permissive amplifier of Wnt signaling rather than a linear dose-dependent ligand.

### Microarray analysis

For microarray analysis of *Rspo3-*WT, *Rspo3*-KO, and *Rspo3*-KI mice, previously published data from our team (GEO GSE145087) [5] were used. The antrum was removed, and total RNA was isolated with TRIzol (Life Technologies) according to the supplier’s protocol using glycogen as coprecipitant. Microarray experiments were performed as independent dual-color dye-reversal color-swap hybridizations using 2 biological replicates. Quality control and quantification of total RNA were assessed using an Agilent 2100 Bioanalyzer (Agilent Technologies) and a NanoDrop 1000 UV–Vis spectrophotometer (Kisker). RNA labeling was performed with a dual-color Quick-Amp Labeling Kit (Agilent Technologies). In brief, mRNA was reverse transcribed and amplified using an oligo-dT-T7 promoter primer, and the resulting cRNA was labeled with cyanine 3–CTP or cyanine 5–CTP. After precipitation, purification, and quantification, 1.25 μg of each labeled cRNA was fragmented and hybridized to whole-genome mouse 4 × 44 K multipack microarrays (Agilent-014868, Whole Mouse Genome 4 × 44 K Microarray Kit) according to the supplier’s protocol (Agilent Technologies). Scanning of microarrays was performed at 5 μm resolution using a G2565CA high-resolution laser microarray scanner (Agilent Technologies) with an extended dynamic range (XDR). Microarray image data were analyzed with Image Analysis/Feature Extraction software G2567AA, version A.11.5.1.1 (Agilent Technologies), using default settings and the GE2_1105_Oct12 extraction protocol. The extracted MAGE-ML files were analyzed further with Rosetta Resolver Biosoftware, build 7.2.2 SP1.31 (Rosetta Biosoftware). Ratio profiles comprising single hybridizations were combined in an error-weighted fashion to create ratio experiments. A 1.5-fold change expression cut-off for ratio experiments was applied together with anticorrelation of dye-swapped ratio profiles, rendering the microarray analysis highly significant (*P* < 0.01), robust, and reproducible [20]. Microarray data have been deposited in the NCBI’s Gene Expression Omnibus (GEO GSE317105).

### GSEA

We performed GSEA on genes ranked by gene expression–based *t* score between gastric corpus epithelium isolated from *Rspo3*-KI or *Rspo3*-KO and *Rspo3-*WT controls using the fgsea R package [21] with 5000 permutations. We used gene sets from Molecular Signature DB version 7.1 [23, 24]. *P* values were adjusted for multiple testing by a global FDR according to the method described by Benjamini and Hochberg [23].

### Single-cell atlas of stomach cell subpopulations

We constructed a human stomach scRNA-seq atlas from published datasets (GSE206785 [25], GSE249874 [26], GSE263018 [27], and from [28]). Each data set was first independently preprocessed using Seurat with doublet identification with scDblFinder [29] followed by integration with Harmony [30]. Global clusters were defined for each data set using known marker genes, and clusters with high proportions of doublets or low-quality cells were flagged for exclusion. Finally, data sets were integrated with Harmony and preprocessed using Seurat. After global identification of major lineages on the combined atlas data set, we performed separate processing and cluster identification for epithelial, stromal, myeloid, and lymphoid cells based on known marker gene expression.

### Gastric cancer transcriptome analysis

Transcriptional data (STAR aligned counts) and clinical annotations for the GDC TCGA Stomach Adenocarcinoma cohort were downloaded from the Xena browser (https://xenabrowser.net/datapages/?cohort=GDC%20TCGA%20Stomach%20Cancer%20(STAD)). Primary cancer samples classified as *Adenomas and Adenocarcinomas* or *Cystic, Mucinous and Serous Neoplasms* were included (n=448 samples). Gene set scores for selected MsigDB Hallmark-sets and for the signatures of the stomach single-cell atlas above were determined using GSVA [31]. Correlations between gene expression levels and/or signatures were determined using Spearman correlation.

### Statistics

Human data were analyzed using IBM SPSS Statistics software, version 30.0.0.0. Differences in CD3+, CD4+, and CD8+ T-cell numbers between RSPO3-low and RSPO3-high tumors were analyzed using the Mann–Whitney U test. Associations of RSPO3 and tumor stage, nodal state, metastatic state, tumor grading, Laurén classification, Ming classification, and UICC classification was tested using the chi-square test (X2-test). For testing of significance, the Fisher's Exact test was used for samples with cell frequencies above 5 in 2×2 contingency tables. The Pearson's Chi-Squared test was used for contingency tables larger than 2×2 and for samples with cell frequencies below 5. Two-tailed testing was performed. Univariate survival analyses were performed according to the Kaplan–Meier method using the log-rank test for the assessment of statistical significance.

Mouse experiments were performed on a minimum of *n* = 3 biological replicates. Microarray analysis was performed using samples from 2 biological replicates. All data are represented as mean ± SEM. Statistics are based on *n* biological replicates. For comparison of 2 groups, a 2-tailed, unpaired *t* test was performed. All analyses of statistical significance were calculated and displayed compared with the reference control group, unless otherwise stated. *P* < 0.05 was considered significant.

Statistical analysis was done using GraphPad Prism software, version 8.3.2, and the IBM SPSS Statistics Software, version 31.0.0.0.

## Results

### Heterogeneous RSPO3 expression across human gastric cancer samples

To gain insight into the role of RSPO3 signaling in human gastric carcinogenesis, we analyzed tissue microarrays generated from primary surgical resections of 277 chemotherapy-naïve patients with gastric adenocarcinoma or adenocarcinoma of the esophagogastric junction (AEG) treated at Charité–Universitätsmedizin Berlin between 1992 and 2004. The cohort included tumors of all stages and is summarized in Supplementary Table 1.

**Fig. 1:**
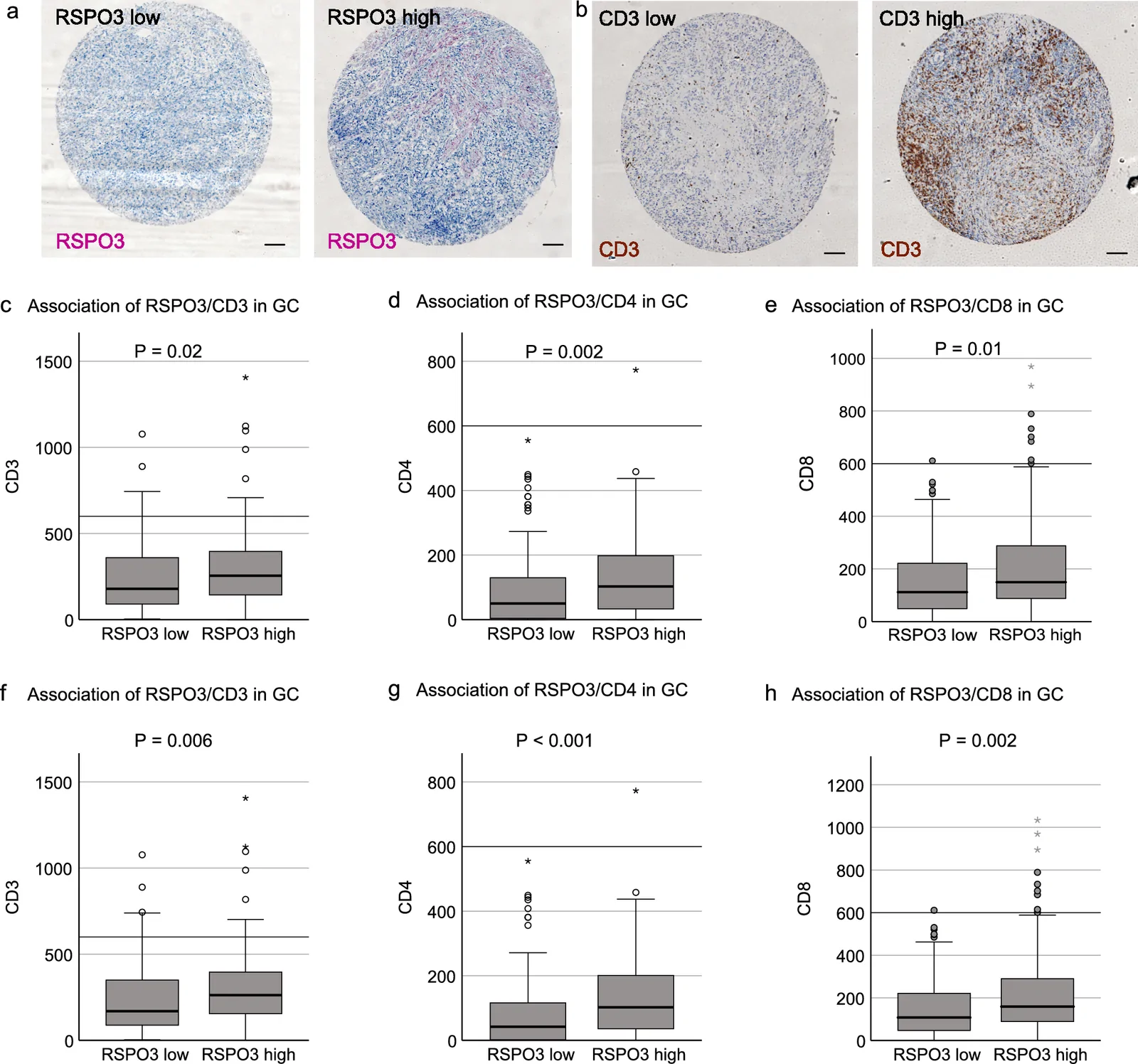
RSPO3-high gastric adenocarcinomas show strong infiltration with T helper cells. **a** In situ hybridization of RSPO3 on tissue microarray of gastric tumor. **b** Immunohistochemistry staining for CD3 on tissue microarrays of gastric tumors. **c**–**e** Box plots visualizing the association of RSPO3 low versus high expression and **c** CD3+ T-cells, **d** CD4+ T-cells, and **e** CD8+ T-cells in gastric tumors, including gastric adenocarcinoma and adenocarcinoma of the esophagogastric junction. **f**–**h** Box plots visualizing the association of RSPO3 low versus high expression and **f** CD3+ T-cells, **g** CD4+ T-cells or **h** CD8+ T-cells in gastric adenocarcinoma. Scale bar = 100 µm

RNAscope analysis demonstrated heterogeneous RSPO3 expression across tumor samples. Based on the predefined scoring criteria, tumors were classified as RSPO3-low (n = 148) or RSPO3-high (n = 129) (Fig. 1a, Supplementary Table 2).

### RSPO3 expression status is not associated with tumor stage or survival

We next examined whether RSPO3 expression was associated with clinicopathological characteristics. Rspo3 expression showed no significant association with tumor stage, nodal status, metastatic status, grading, Laurén classification, Ming classification, or UICC stage (Supplementary Fig. 1a–g). Likewise, Kaplan–Meier analysis revealed no difference in overall survival between patients with RSPO3-low and RSPO3-high tumors (Supplementary Fig. 1h).

Subgroup analyses performed separately for gastric adenocarcinoma and AEG similarly showed no association between RSPO3 expression and clinicopathological parameters or survival (Supplementary Figs. 2 and 3).

### RSPO3-high tumors show high T cell infiltration

As RSPO3 expression was independent of tumor stage, we hypothesized that it reflects differences in tumor biology rather than disease progression. Because our previous studies identified Rspo3 as a regulator of epithelial inflammatory responses [7, 8], we analyzed T-cell infiltration by immunohistochemistry for CD3, CD4, and CD8 (Fig. 1b).

Quantitative analysis demonstrated significantly higher numbers of infiltrating CD4+ helper T cells and CD8+ cytotoxic T cells in RSPO3-high tumors (Fig. 1c–e). Subgroup analysis revealed that this association was confined to gastric adenocarcinoma and was not detected in AEG (Fig. 1f–h, Supplementary Fig. 4a–c), although the limited number of AEG cases may have reduced statistical power. Importantly, multivariable analysis confirmed that the association between RSPO3 expression and T-cell infiltration remained significant after adjustment for clinicopathological variables (Supplementary Fig. 5a).

### RSPO3 positively associates with PD-L1 expression in gastric adenocarcinoma

**Fig. 2:**
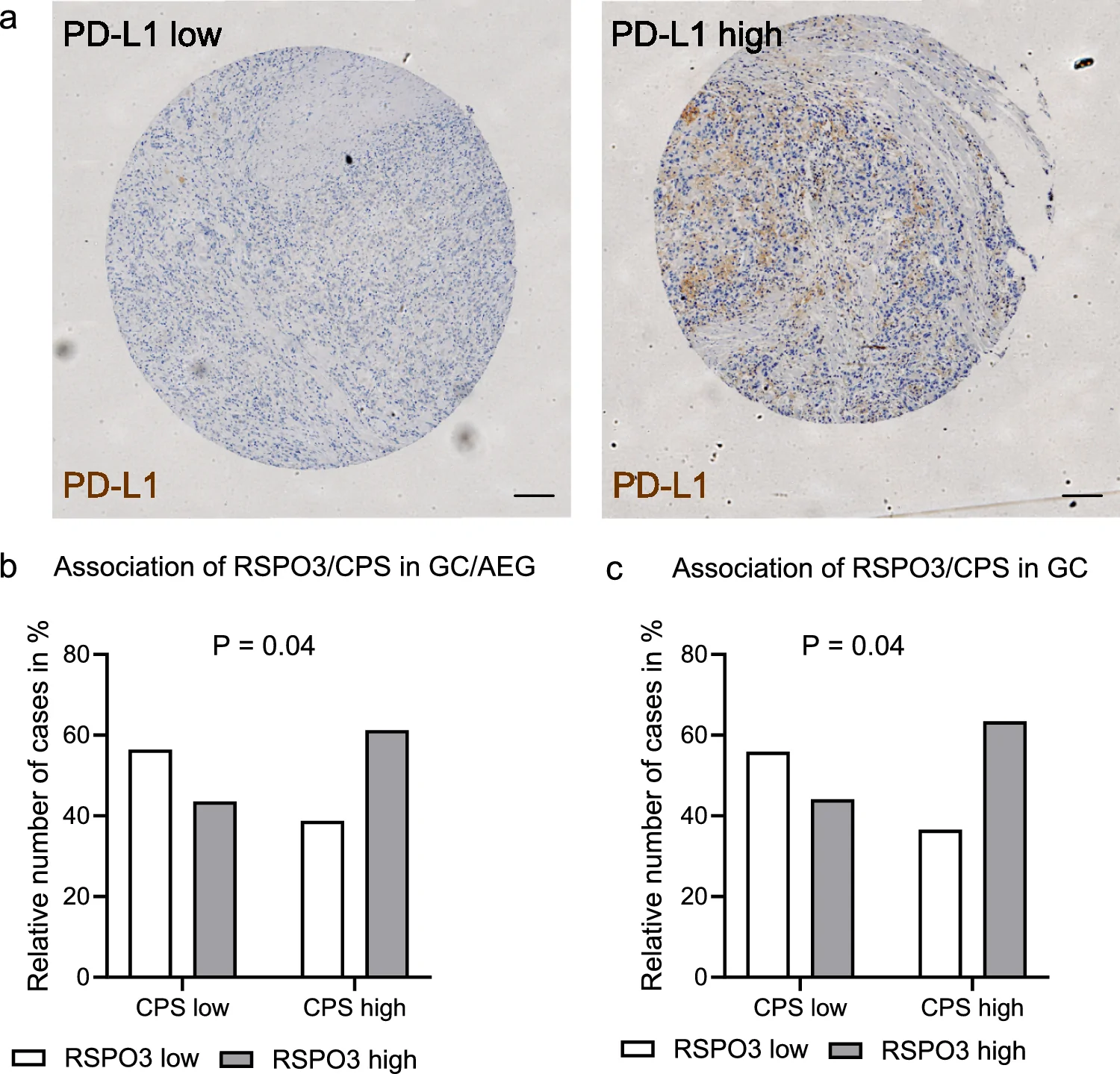
RSPO3 expression associates with PD-L1 positivity in gastric adenocarcinoma. **a** Immunohistochemistry staining for PD-L1 on tissue microarrays of gastric tumors. **b** + **c** Bar chart visualizing the association of RSPO3 low versus high expression and CPS in gastric tumors, including **B** gastric adenocarcinoma and adenocarcinoma of the esophagogastric junction and **C** gastric adenocarcinoma only. Scale bar = 100 µm

Given the increased T-cell infiltration in RSPO3-high tumors, we next investigated expression of the immune checkpoint molecule PD-L1, an established predictive biomarker for immunotherapy in gastric cancer [32]. PD-L1 immunohistochemistry was performed, and the combined positive score (CPS) was calculated (Fig. 2a).

RSPO3-high tumors exhibited significantly higher CPS values than RSPO3-low tumors (Fig. 2b). Similar to the findings for T-cell infiltration, this association was observed in gastric adenocarcinoma (Fig. 2c) but not in AEG (Supplementary Fig. 4d). Multivariable analysis confirmed that the association between RSPO3 expression and PD-L1 remained independently significant (Supplementary Fig. 5b).

### *Rspo3* overexpression alone does not induce T cell infiltration or PD-L1 expression in the murine stomach.

To determine whether Rspo3 directly regulates immune infiltration, we first analyzed mice with conditional stromal *Rspo3* overexpression. Following two months of *Rspo3* induction, gastric tissues were analyzed for CD3 and PD-L1 expression.

Despite robust *Rspo3* overexpression, neither CD3+ T-cell infiltration nor PD-L1 expression was increased compared with control mice (Supplementary Fig. 6), indicating that Rspo3 alone is insufficient to induce these immune responses.

### Rspo3 promotes T-cell infiltration and PD-L1 expression during *H. pylori* infection in mice

**Fig. 3:**
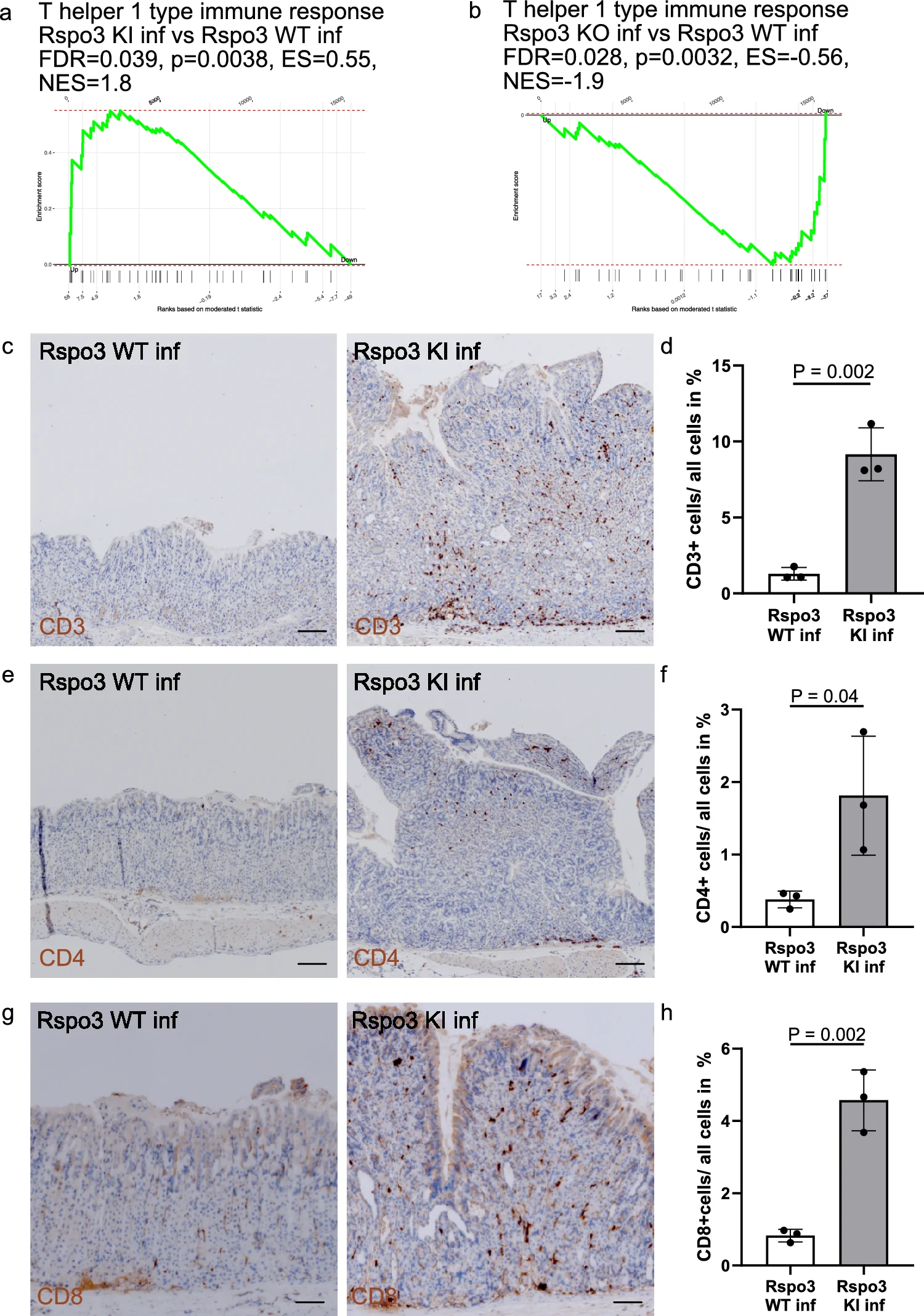
Rspo3 promotes infiltration of T helper type 1 cells into the mucosa of *Helicobacter pylori*-infected mice. **a **+ **b** Gene set enrichment analysis comparing the expression of genes assigned to T helper type 1 immune response in 2 months *H. pylori*-infected **A** Rspo3 overexpressing (Rspo3 KI) versus Rspo3 WT mice, and **B** Rspo3 knockout (Rspo3 KO) versus Rspo3 WT mice **c**, **e**, **g** immunohistochemistry staining for **c** CD3, **e** CD4, **g** CD8 on gastric tissue of 2 months *H. pylori*-infected Rspo3 WT and Rspo3 KI mice **d**, **f**, **h** quantification of **d** CD3+, **f** CD4+, **h** CD8+ cells per all mucosal cells in 2 months *H. pylori*-infected Rspo3 WT and Rspo3 KI mice. Scale bar = 100 µm

Because gastric carcinogenesis typically develops in the setting of chronic *H. pylori* infection [33], we next investigated infected *Rspo3*-overexpressing mice, which develop precancerous lesions that are characterized by chronic inflammation, epithelial hyperplasia, metaplasia, and the development of adenoma [5]. Transcriptome analysis followed by GSEA demonstrated significant enrichment of Th1-associated gene signatures in infected *Rspo3*-overexpressing mice compared with infected wild-type animals (Fig. 3a).

To confirm these findings, we analyzed conditional *Rspo3* knockout mice. In contrast to overexpression, deletion of *Rspo3* significantly reduced Th1-associated gene signatures despite persistent *H. pylori* infection (Fig. 3b), demonstrating that Rspo3 is a major regulator of infection-associated T-helper responses.

Immunohistochemical analyses confirmed significantly increased infiltration of CD3+, CD4+, and CD8+ T cells in infected *Rspo3*-overexpressing mice compared with infected controls (Fig. 3c–h).

**Fig. 4:**
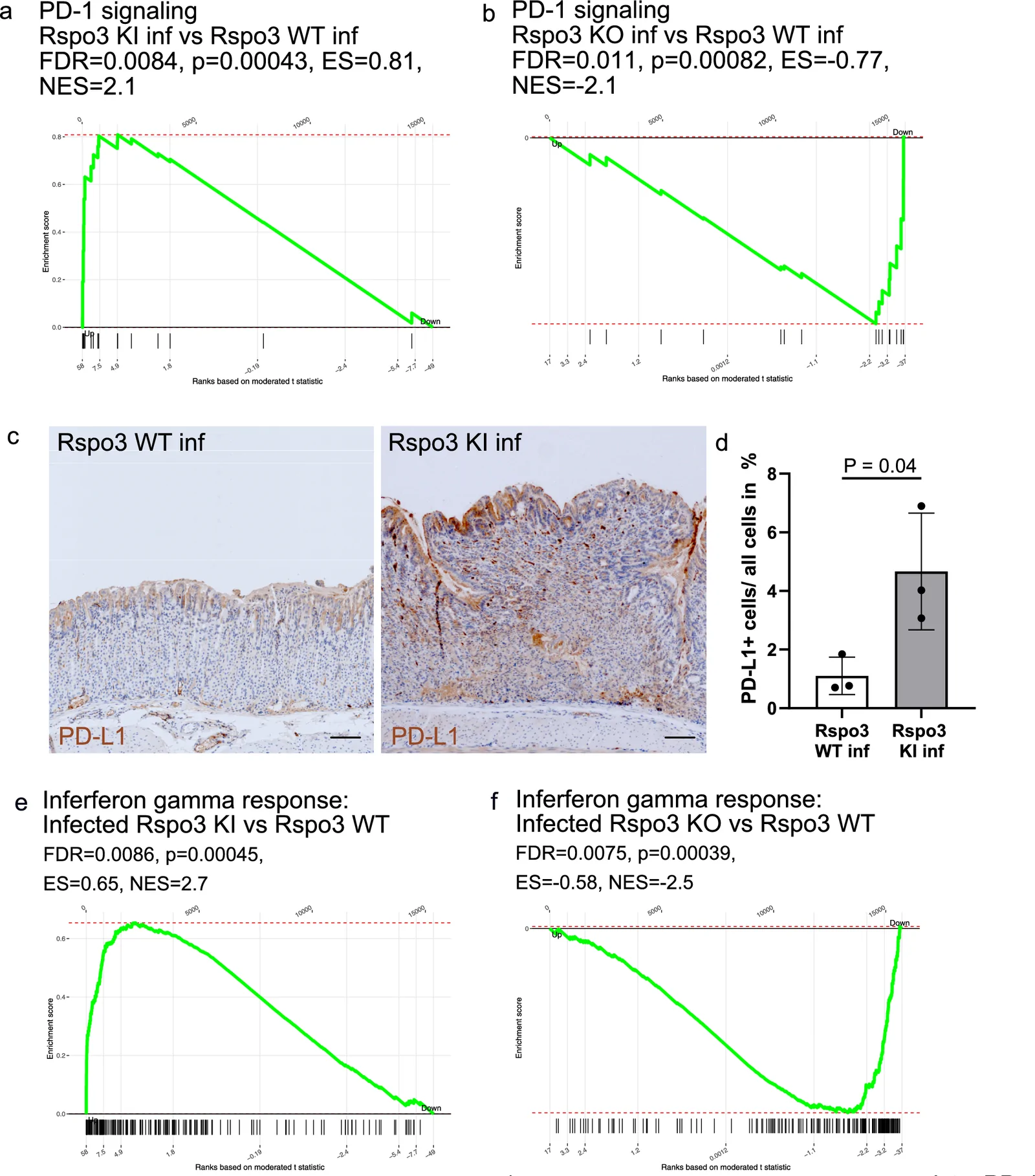
Rspo3 promotes PD-L1 expression in mice. **a** + **b** Gene set enrichment analysis comparing the expression of genes assigned to PD-1 signaling in 2 months *H. pylori*-infected **a** Rspo3 overexpressing (Rspo3 KI) versus Rspo3 WT mice, and **b** Rspo3 knockout (Rspo3 KO) versus Rspo3 WT mice **c** Immunohistochemistry staining for PD-L1 on gastric tissue of 2 months *H. pylori*-infected Rspo3 WT and Rspo3 KI mice **d** Quantification of PD-L1+ cells per all mucosal cells in 2 months *H. pylori*-infected Rspo3 WT and Rspo3 KI mice. **e** + **f** Gene set enrichment analysis comparing the expression of genes assigned to Interferon gamma response in 2 months *H. pylori*-infected **e** Rspo3 overexpressing (Rspo3 KI) versus Rspo3 WT mice, and **f** Rspo3 knockout (Rspo3 KO) versus Rspo3 WT mice Scale bar = 100 µm

We next investigated immune checkpoint signaling. GSEA demonstrated enrichment of PD-1 signaling genes in infected *Rspo3*-overexpressing mice and depletion following *Rspo3* deletion (Fig. 4a, b). Consistent with these transcriptomic changes, PD-L1 protein expression was significantly increased in infected *Rspo3*-overexpressing mice (Fig. 4c, d).

Because both PD-L1 expression and T-cell activation are regulated by Interferon-gamma (IFN-γ), we also analyzed IFN-γ signaling. IFN-γ response signatures were significantly enriched in infected *Rspo3*-overexpressing mice but reduced following *Rspo3* deletion (Fig. 4e, f).

Together, these findings demonstrate that Rspo3 promotes Th1-associated immune infiltration and PD-L1 expression specifically in the context of *H. pylori*-induced gastric inflammation.

### Rspo3 regulates T cell infiltration and PD-L1 expression via epithelial LGR4/5 receptors

**Fig. 5:**
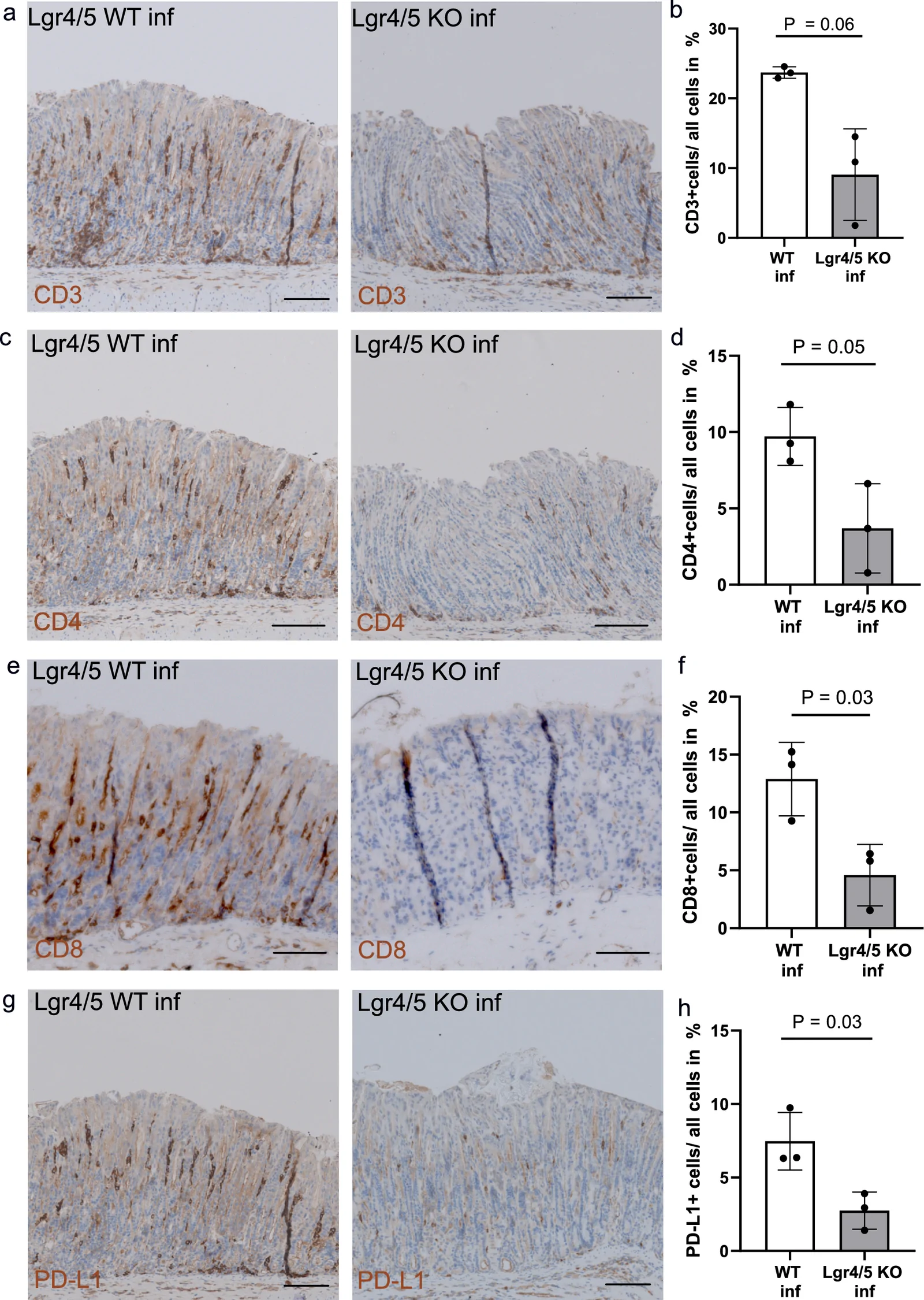
Rspo3-dependent immune cell infiltration and PD-L1 expression are regulated via the LGR4/5 receptor. **a**, **c**, **e**, **g** Immunohistochemistry staining for **a** CD3, **c** CD4, **e** CD8, **g** PD-L1 on gastric tissue of 2 months *H. pylori*-infected Lgr4/5 WT and Lgr4/5 KO mice. **b**, **d**, **f**, **h** Quantification of **b** CD3+, **d** CD4+, **f** CD8+, **h** PD-L1+ cells per all mucossal cells in 2 months *H. pylori*-infected Lgr4/5 WT and Lgr4/5 KO mice. Scale bar = 100 µm

We and others have reported that epithelial cells can act as sensors and regulators of inflammatory signals [7, 8]. To determine whether stromal *Rspo3* regulates immune infiltration via epithelial responses, we analyzed mice with an epithelial-specific knockout of the *Rspo3* receptors *Lgr4* and *Lgr5*. Following *H. pylori* infection, *Lgr4/5*-deficient mice exhibited significantly fewer CD3⁺, CD4⁺, and CD8⁺ T cells than infected wild-type controls (Fig. 5a–f). PD-L1-positive cells were likewise significantly reduced (Fig. 5g, h).

These findings indicate that *Rspo3*-dependent regulation of T-cell infiltration and PD-L1 expression requires epithelial LGR4/LGR5 signaling.

### RSPO3 expression specifically correlates with immune cell infiltration in intestinal-type gastric cancer in a TCGA cohort

To validate our observations, we analyzed the TCGA gastric adenocarcinoma cohort [10]. Consistent with our patient cohort, RSPO3 expression positively correlated with CD3 and CD4 expression (Supplementary Fig. 7a,b).

**Fig. 6:**
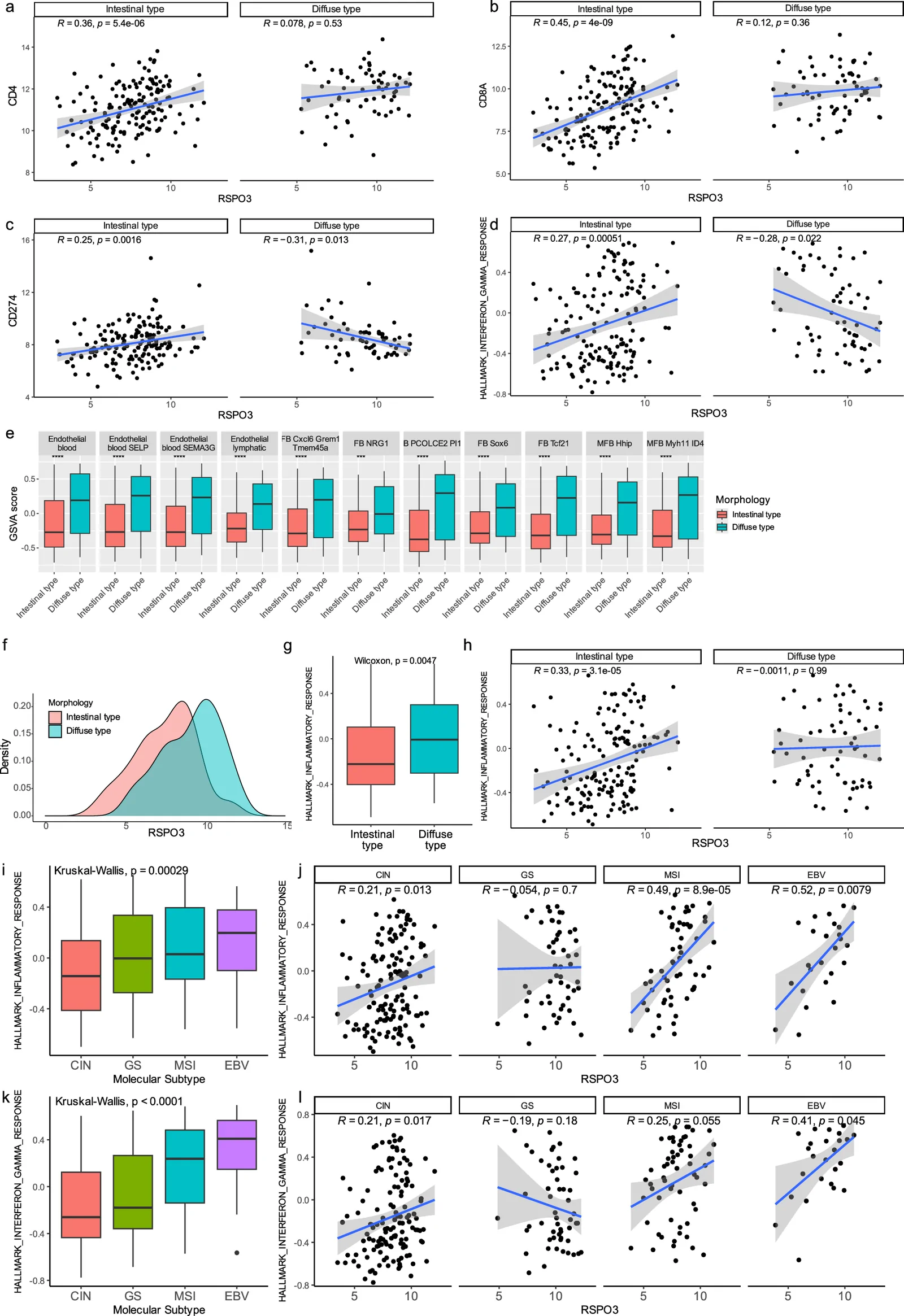
RSPO3 specifically correlates with inflammation in intestinal type GC in TCGA cohort. **a**–**d** Scatter plots depicting the correlation between RSPO3 expression and **a** CD4, **b** CD8, **c** PD-L1, and **d** the gene. siganture for IFNg response in intestinal-type vs diffuse-type gastric adenocarcinoma of The Cancer genome Atlas. (TCGA) cohort. **e** Box plots showing the abundance of different stromal cell signatures in intestinal-type vs diffuse-type gastric. adenocarcinoma of TCGA cohort. **f** Expression density of RSPO3 in intestinal-type vs diffuse-type gastric adenocarcinoma of TCGA cohort. **g** Expression intensity of genes associated with inflammatory response in intestinal-type vs diffuse-type gastric. Adenocarcinoma of TCGA cohort. **h** Scatter plots depicting the correlation between RSPO3 expression and expression intensity of genes associated with. Inflammatory response in intestinal-type vs diffuse-type gastric adenocarcinoma of TCGA cohort. **i** Expression intensity of genes associated with inflammatory response in the four molecular subtypes of gastric. Adenocarcinoma in the TCGA cohort. CIN = chromosomal instability, GS = genomically stable, MSI = microsatellite. Instability, EBV = Epstein–Barr Virus positive. **j** Scatter plots depicting the correlation between RSPO3 expression and expression intensity of genes associated with. Inflammatory response in the four molecular subtypes of gastric adenocarcinoma in the TCGA cohort. **k** Expression intensity of genes associated with IFNg response in the four molecular subtypes of gastric. Adenocarcinoma in the TCGA cohort. CIN = chromosomal Instability, GS = genomically stable, MSI = microsatellite. instability, EBV = Epstein–Barr Virus positive. **l** Scatter plots depicting the correlation between RSPO3 expression and expression intensity of genes associated with. IFNg response in the four molecular subtypes of gastric adenocarcinoma in the TCGA cohort

Subtype analyses revealed that these correlations were restricted to intestinal-type gastric cancer. In intestinal-type tumors, RSPO3 expression positively correlated with CD3, CD4, and CD8 expression, Th1 signatures, CD8⁺ T-cell signatures, PD-L1 expression, and IFN-γ signaling, whereas these correlations were not observed in diffuse-type tumors (Fig. 6a–d, Supplementary Fig. 7).

Further analyses showed that diffuse-type gastric cancers contained significantly higher stromal cell abundance, higher RSPO3 expression, and greater immune infiltration than intestinal-type tumors (Fig. 6e–h, Supplementary Fig. 8), which is in line with observations made in previous publications [34, 35]. However, in contrast to intestinal-type tumors, inflammatory signatures in diffuse-type cancers were independent of RSPO3 expression Fig. 6h, Supplementary Fig. 8c, e).

To further validate our findings, we correlated RSPO3 expression and inflammatory response in the different TCGA subtypes. We observe positive correlations between RSPO3 expression and T-cell infiltration, inflammatory response, and IFN-γ signaling in the in the molecular subtypes chromosomal instability (CIN), microsatellite instability (MSI), and Epstein–Barr virus positive (EBV), whereas these correlations were absent in the genomically stable subtype (Fig. 6i–l, Supplementary Fig. 9a-f), although RSPO3 expression is on average the highest in this subtype (Supplementary Fig. 9g). The genomically stable subtype of gastric cancer strongly overlaps with the histopathological diffuse type GC, being in line with our observations that correlations are found for intestinal but not diffuse type GC.

## Discussion

We demonstrate that stromal RSPO3 expression is associated with the immune phenotype of gastric adenocarcinoma. High RSPO3 expression correlates with increased T-cell infiltration, PD-L1 expression, and IFN-γ signaling, specifically in intestinal-type gastric cancer.

Using complementary conditional mouse models, we provide mechanistic evidence that stromal *Rspo3* regulates immune responses through epithelial LGR4/LGR5 receptors. The *H. pylori* infection model combined with epithelial *Lgr4/5* deletion reflects early stages of gastric carcinogenesis and demonstrates a causal role for epithelial LGR4/LGR5 signaling in regulating immune responses during early disease development. In contrast, the *Rspo3* overexpression model recapitulates later precancerous changes characterized by metaplasia and adenoma formation, demonstrating that dysregulated RSPO3–LGR4/LGR5 signaling contributes to the development of precancerous lesions. Together, these models support a causal role for RSPO3–LGR4/LGR5 signaling in shaping the gastric tumorigenic microenvironment. However, the associations observed in the human cohort remain correlative and should be interpreted within the temporal context of gastric cancer development.

Our data indicate that both the stromal cell microenvironment as well as the immune landscape show significant differences between diffuse and intestinal-type GC, which likely reflects the fact that cancer initiation and progression trajectories are fundamentally different between the two subtypes. LGR5 is expressed at significantly lower levels in diffuse-type gastric cancer compared to intestinal-type GC [36, 37]. As we show that RSPO3-driven immune cell infiltration is dependent on epithelial LGR4/5 receptors, the RSPO-LGR axis may act differently in diffuse-type GC compared to intestinal-type GC, which represents one possible reason for the differences observed in tumor samples in our study.

Previous studies reported positive correlations between RSPO3 expression and infiltration of NK cells, dendritic cells, and T cells across several tumor entities [38]. Our data extend these findings to gastric cancer and identify an epithelial mechanism underlying this association. While *Rspo3* has been proposed to directly recruit immune cells via Lgr6 expressed on NK and T cells [38], our results indicate that epithelial LGR4/5 signaling is required for *Rspo3*-dependent immune infiltration. We have previously shown that the RSPO3-LGR4/5 axis activates epithelial NF-κB signaling and innate immune responses during *H. pylori* infection [8]. The present study extends these observations by demonstrating that *Rspo3* promotes adaptive immune responses in precancerous lesions and is associated with inflammatory features in human gastric cancer. The high prevalence of RSPO3-high tumors (46.6%) further suggests that RSPO3-dependent inflammation represents a common feature of gastric tumorigenesis.

As the majority of gastric cancers are attributable to *H. pylori* [33], the relationship between RSPO3 signaling and infection-associated inflammation is of particular interest. Eradication of *H. pylori* after endoscopic resection reduces gastric cancer recurrence [39] and is accompanied by decreased CD3+ cell numbers and lower PD-L1+/CD4+ ratios in patients who do not develop metachronous disease [40]. These findings raise the possibility that modulation of RSPO3 signaling could influence the inflammatory tumor microenvironment and therapeutic responses. Because our cohort predates the introduction of immune checkpoint inhibitors, we could not assess whether RSPO3 predicts immunotherapy response. Future studies should determine whether RSPO3-high tumors derive greater benefit from checkpoint inhibition and whether combined targeting of RSPO3 and immune checkpoints provides a therapeutic advantage.

To our knowledge, this is the first study to systematically analyze the expression of RSPO3 in gastric cancer. Nonetheless, our study has several limitations. First of all, it was performed on the basis of tissue microarrays (TMAs), which, like biopsies, constitute only small parts of potentially heterogeneous tumor lesions. Although the average scores of two cores per tumor were included to improve representativeness, TMAs cannot completely reflect the spatial complexity of the tumor microenvironment. A larger follow-up study investigating the relationship of RSPO3 and immune response in a higher number of biopsies per tumor, on whole-slide sections or by digital spatial profiling may provide a more comprehensive characterization of the tumor immune microenvironment and further validate our observations. Second, only 44 patients with adenocarcinoma of the esophagogastric junction were included, limiting statistical power for subgroup analyses. To exclude bias caused by the heterogeneous group, we have analyzed both subgroups independently. Validation in larger independent AEG cohorts is therefore warranted. Third, our clinical cohort lacked information on premalignant lesions, precluding direct assessment of RSPO3 during early human gastric carcinogenesis. However, the corresponding observations in our *H. pylori* mouse models suggest that *Rspo3*-driven inflammation is established before malignant transformation. Fourth, our cohort does not contain information on the *H. pylori* infection status. However, as 93% of gastric cancer cases in Europe are attributable to *H. pylori *[33], we assume that the majority of the patients included in our cohort were also *H. pylori* positive.

In summary, our study identifies stromal RSPO3 as a marker of an inflamed, PD-L1-positive tumor microenvironment in intestinal-type gastric cancer and provides mechanistic evidence that this phenotype is mediated through epithelial LGR4/5 signaling during *H. pylori*-associated gastric carcinogenesis. Future studies are needed to assess whether the RSPO3-dependent inflammatory tumor microenvironment determines response to immunotherapy.

## Supplementary Information

Below is the link to the electronic supplementary material.

Supplementary file1 (PDF 3467 kb)
