# Supplementary material for: Mesenchymal R-spondin 3 promotes an immunogenic tumor microenvironment with adaptive immune resistance in human gastric adenocarcinoma
Source: Gastric Cancer. 2026 Aug 24;29(6):1216–29. doi: 10.1007/s10120-026-01791-2 (PMC13755003; doi:10.1007/s10120-026-01791-2)
Supplement: Supplementary file 1 [file 10120_2026_1791_MOESM1_ESM.pdf]

Supplemental Figure 1: The RSPO3 expression status is not associated with tumor stage or survival in gastric tumors.

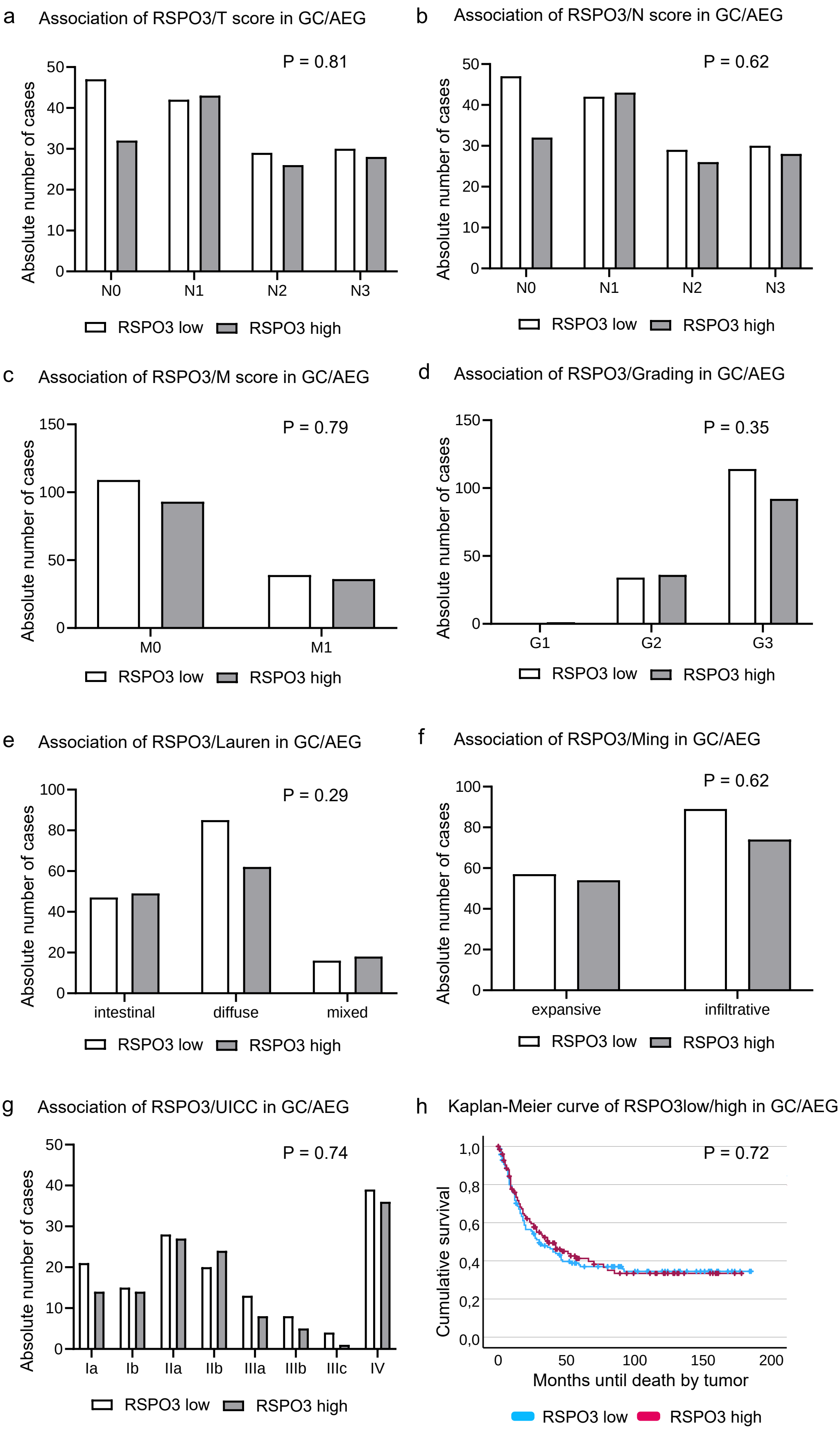

a-g) Bar chart visualizing the association between high versus low RSPO3 expression and a) T score, b) nodal status, c) metastatic status, d) Grading, e) Laurén classification, f) Ming classification, g) UICC classification in gastric tumors, including gastric adenocarcinoma and adenocarcinoma of the esophagogastric junction

h) Kaplan-Meier curve showing survival of patients with RSPO3 low (blue) versus RSPO3 high (red) gastric tumors, including gastric adenocarcinoma and adenocarcinoma of the esophagogastric junction

Supplementary Figure 2: The RSPO3 expression status is not associated with tumor stage or survival in gastric adenocarcinoma (GC).

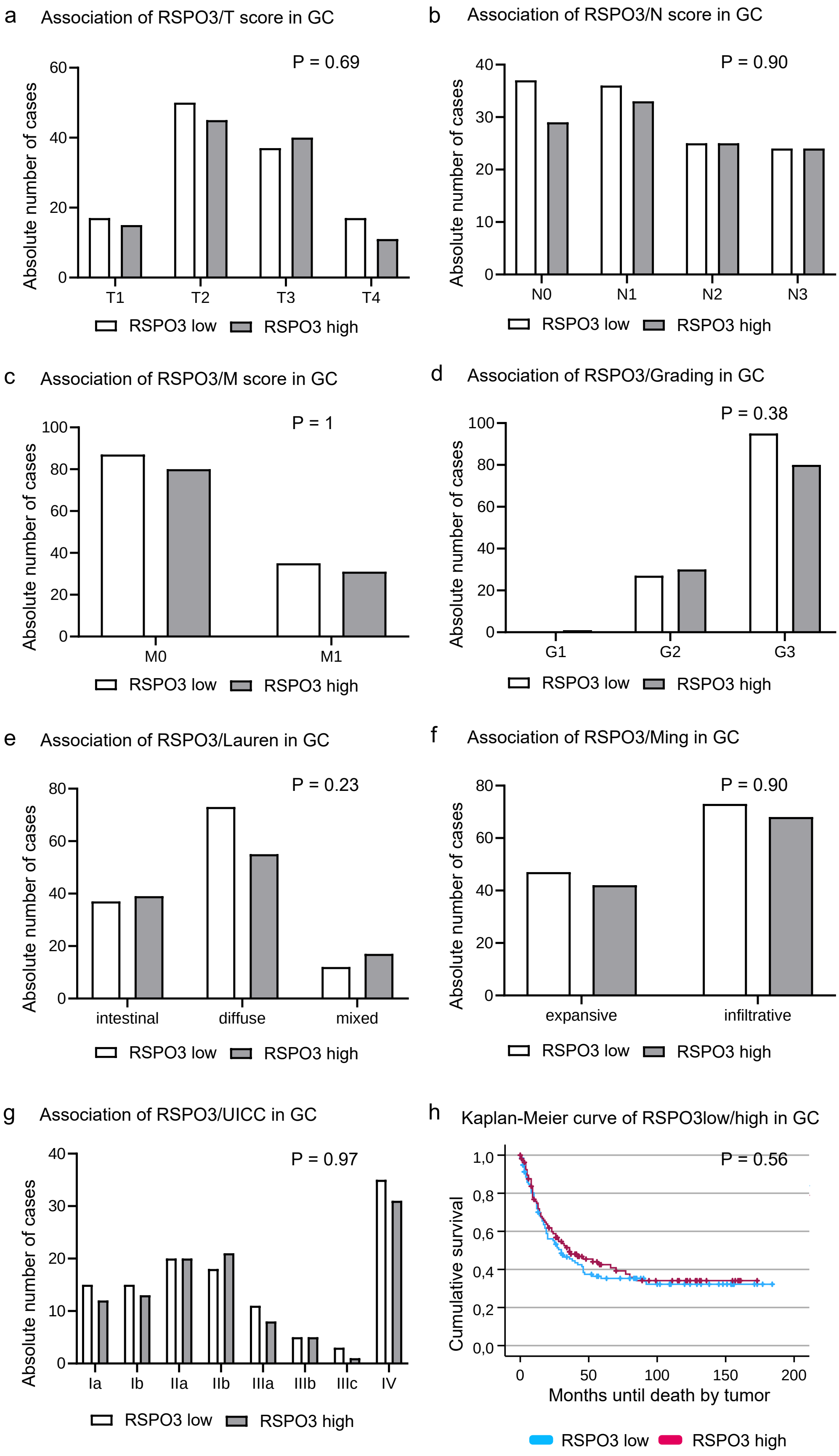

a-g) Bar chart visualizing the association between high versus low RsSPO3 expression and a) T score, b) nodal status, c) metastatic status, d) Grading, e) Laurén classification, f) Ming classification, g) UICC classification in gastric adenocarcinoma

h) Kaplan-Meier curve showing survival of patients with RSPO3 low (blue) versus RSPO3 high (red) gastric adenocarcinoma

Supplementary Figure 3: The RSPO3 expression status is not associated with tumor stage or survival in adenocarcinoma of the esophagogastric junction.

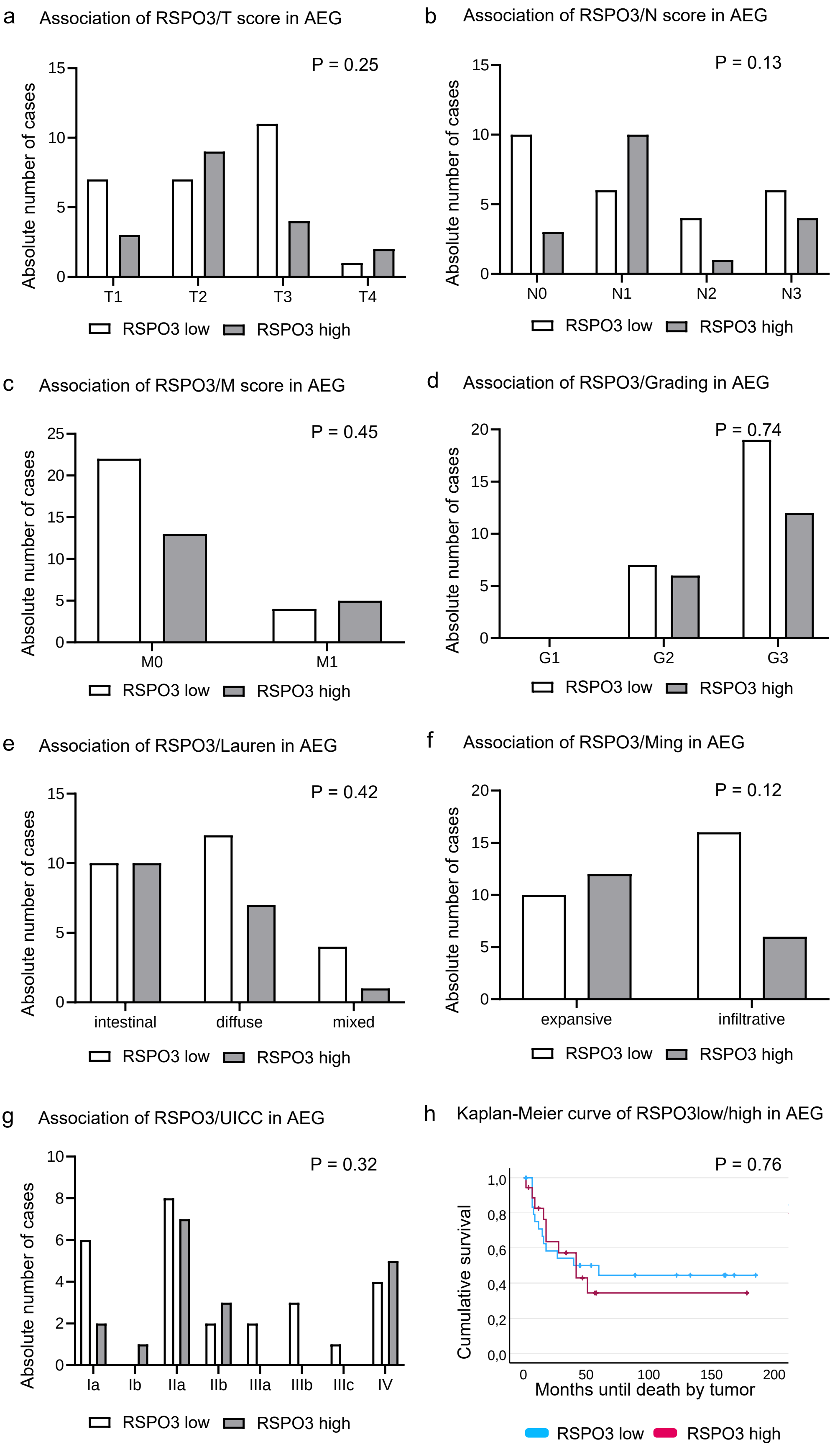

a-g) Bar chart visualizing the association between high versus low RSPO3 expression and a) T score, b) nodal status, c) metastatic status, d) Grading, e) Laurén classification, f) Ming classification, g) UICC classification in adenocarcinoma of the esophagogastric junction

h) Kaplan-Meier curve showing survival of patients with RSPO3 low (blue) versus RSPO3 high (red) adenocarcinoma of the esophagogastric junction

Supplementary Figure 4: Association between RSPO3 and immune cell infiltration can not be confirmed in AEG tumors

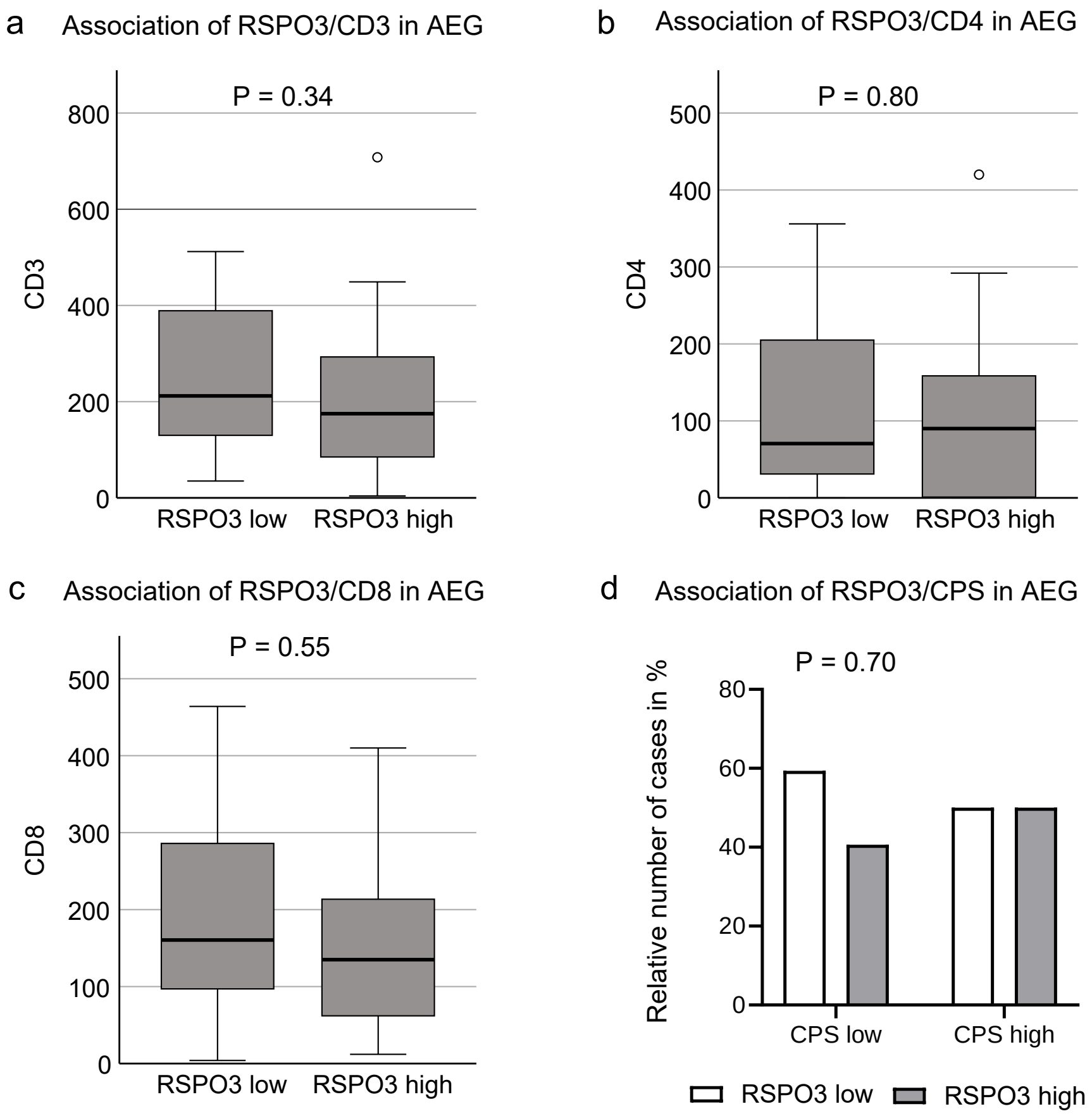

a-c) Box plots visualizing the association of RSPO3 low versus high expression and a) CD3+ T-cells, b) CD4+ T-cells, c) CD8+ T-cells in adenocarcinoma of the esophagogastric junction (AEG tumors).

d) Bar chart visualizing the correlation of RSPO3 low versus high expression and CPS in adenocarcinoma of the esophagogastric junction (AEG tumors).

Supplementary Figure 5: Multivariable analysis

a

Variables in the Equation

		B	S.E.	Wald	df	Sig.	Exp(B)
Step 1[a]	RSPO3	1,103	,316	12,166	1	<,001	3,014
	Age	,450	,320	1,970	1	,160	1,568
	Sex	-,199	,325	,374	1	,541	,820
	Lauren	,023	,238	,009	1	,924	1,023
	pT	,120	,207	,336	1	,562	1,127
	pN	-,651	,391	2,775	1	,096	,522
	pM	-1,064	,414	6,594	1	,010	,345
	Constant	-,464	,776	,357	1	,550	,629

b

Variables in the Equation

		B	S.E.	Wald	df	Sig.	Exp(B)
Step 1[a]	RSPO3	,824	,371	4,933	1	,026	2,280
	Age	,166	,374	,196	1	,658	1,180
	Sex	-,615	,411	2,247	1	,134	,540
	Lauren	-,013	,286	,002	1	,965	,988
	pT	,024	,231	,011	1	,918	1,024
	pN	-,624	,436	2,044	1	,153	,536
	pM	-,590	,505	1,364	1	,243	,555
	Constant	-,613	,903	,462	1	,497	,541

a+ b) Multivariable analysis for a) CD4, and b) CPS

Supplementary Figure 6: Rspo3 does not promote T cell infiltration or PD-L1 expression during homeostasis in mice.

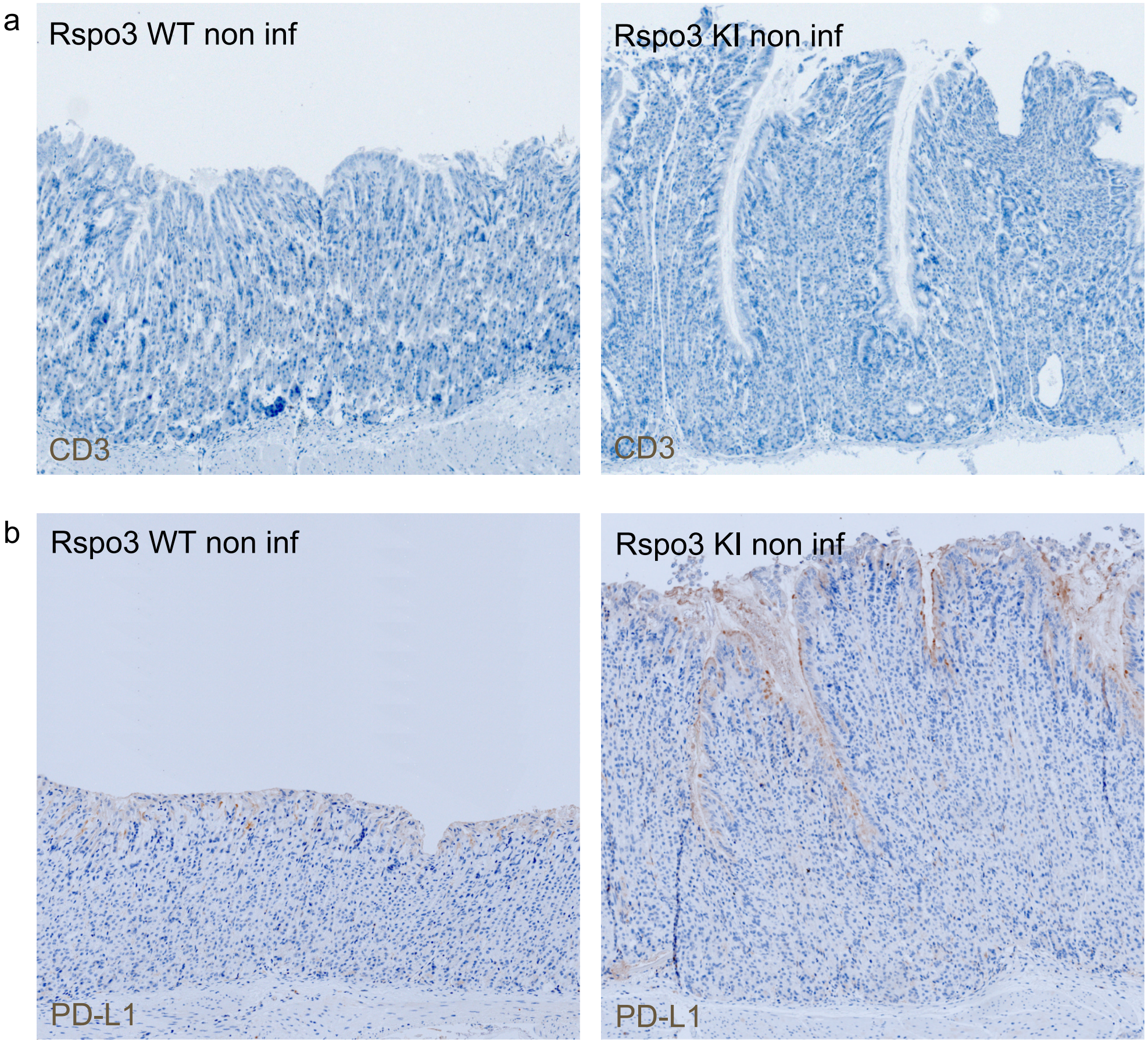

a+b) Immunohistochemistry staining for A) CD3 and B) PD-L1 on gastric tissue of non infected Rspo3 WT and Rspo3 overexpressing (Rspo3 KI) mice

Supplementary Figure 7: RSPO3 is associated with T cell infiltration

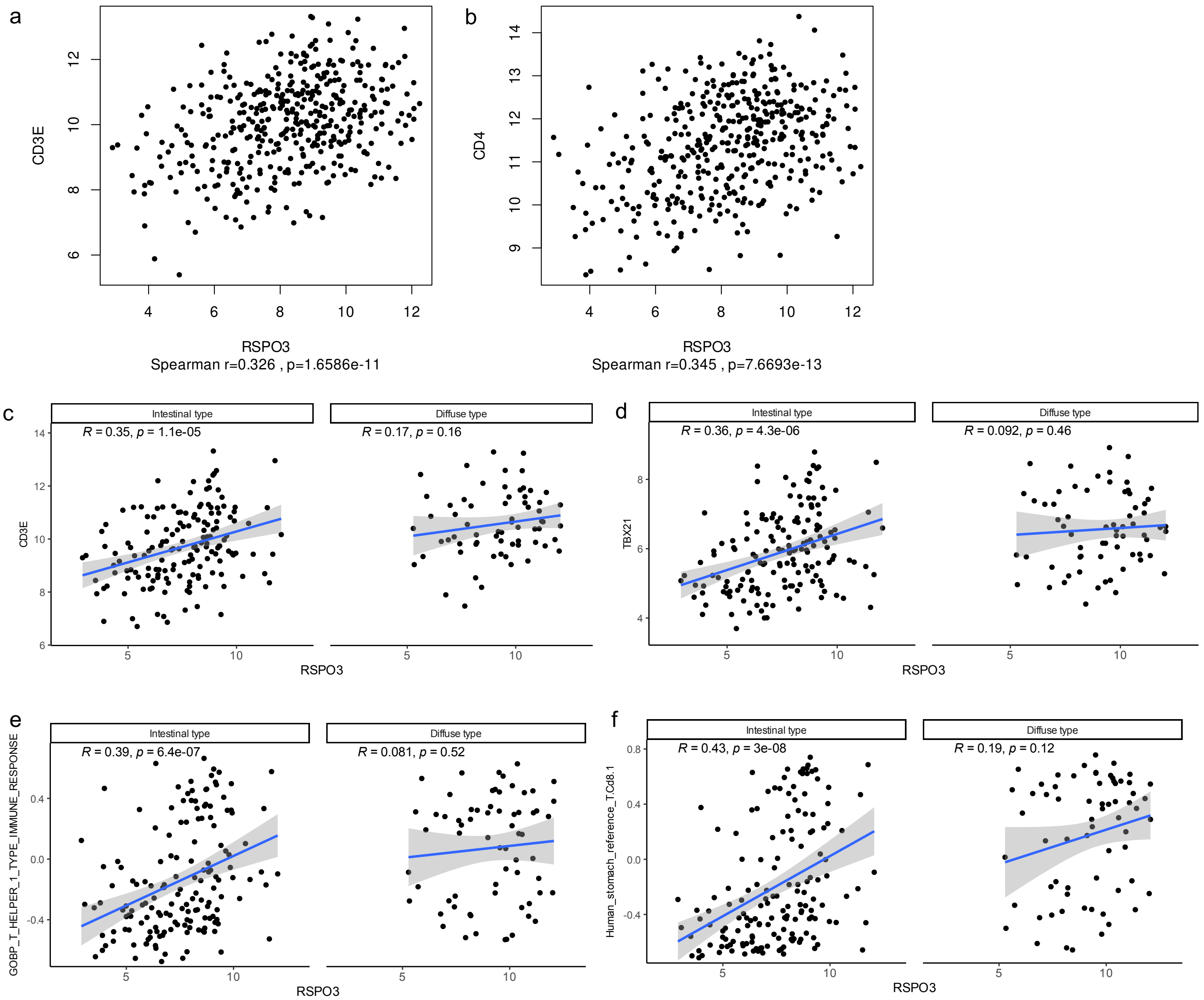

a,b) Scatter plot demonstrating the correlation between RSPO3 expression and a) CD3 and b) CD4 expression in gastric adenocarcinoma of The Cancer genome Atlas cohort.

c-f) Scatter plots depicting the correlation between Rspo3 expression and c) CD3, d) Tbet, e) the gene signature of T helper type 1 immune response, and f) the gene siganture for CD8+ T-cells in intestinal-type vs diffuse-type gastric adenocarcinoma of The Cancer genome Atlas (TCGA)cohort.

Supplementary Figure 8: Immunity in intestinal-type vs diffuse-type GC

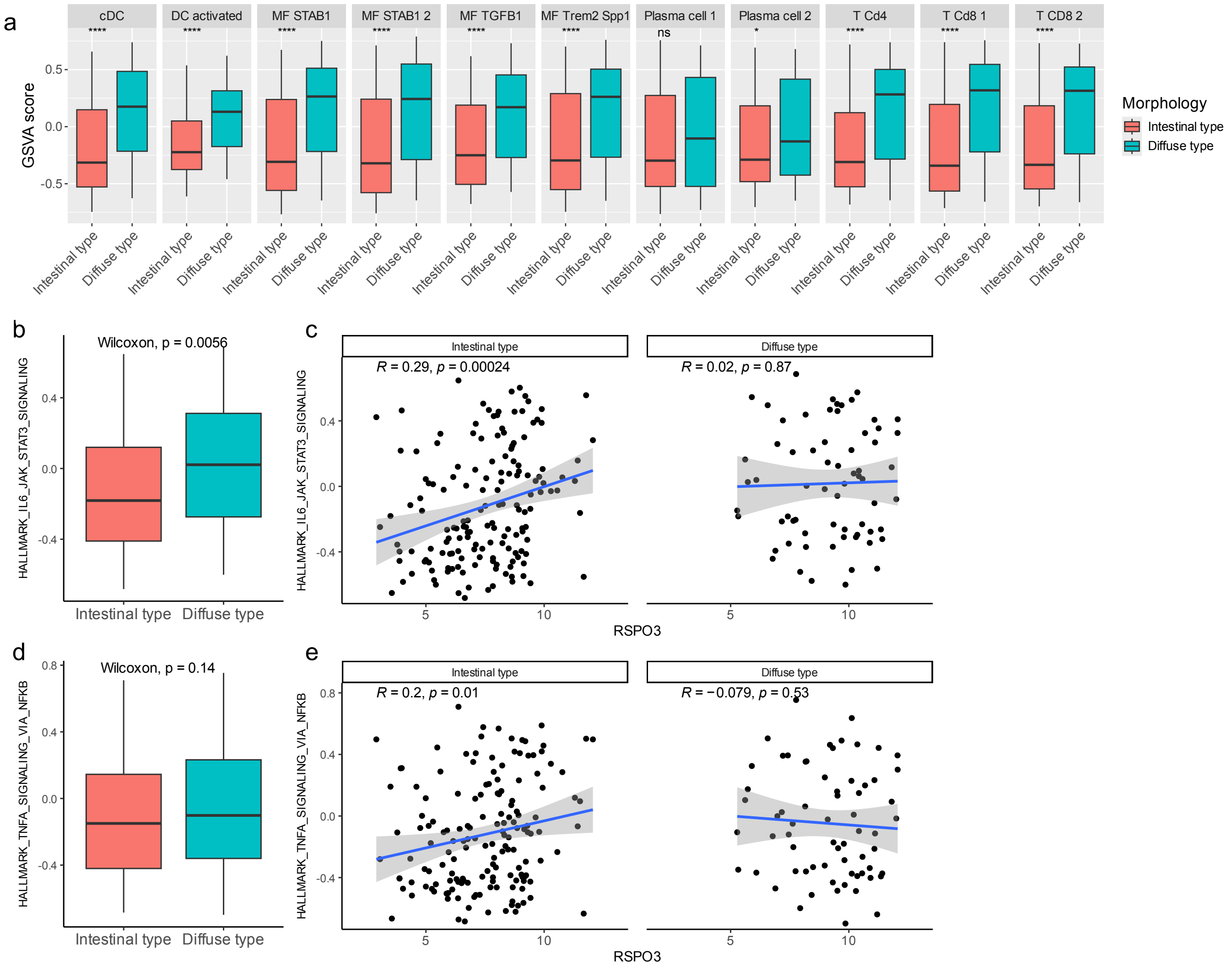

a) Box plots showing the abundance of different immune cell signatures in intestinal-type vs diffuse-type gastric adenocarcinoma of TCGA cohort.

b,d) Expression intensity of genes associated with b) IL6/JAK/STAT3 signaling and d) TNFa signaling in intestinal-type vs diffuse-type gastric adenocarcinoma of TCGA cohort.

c,e) Scatter plots depicting the correlation between Rspo3 expression and expression intensity of genes associated with c) IL6/JAK/STAT3 signaling and e) TNFa signaling in intestinal-type vs diffuse-type gastric adenocarcinoma of TCGA cohort.

Supplementary Figure 9: Correlation between RSPO3 and immune cell infiltration in different molecular subtypes of GC

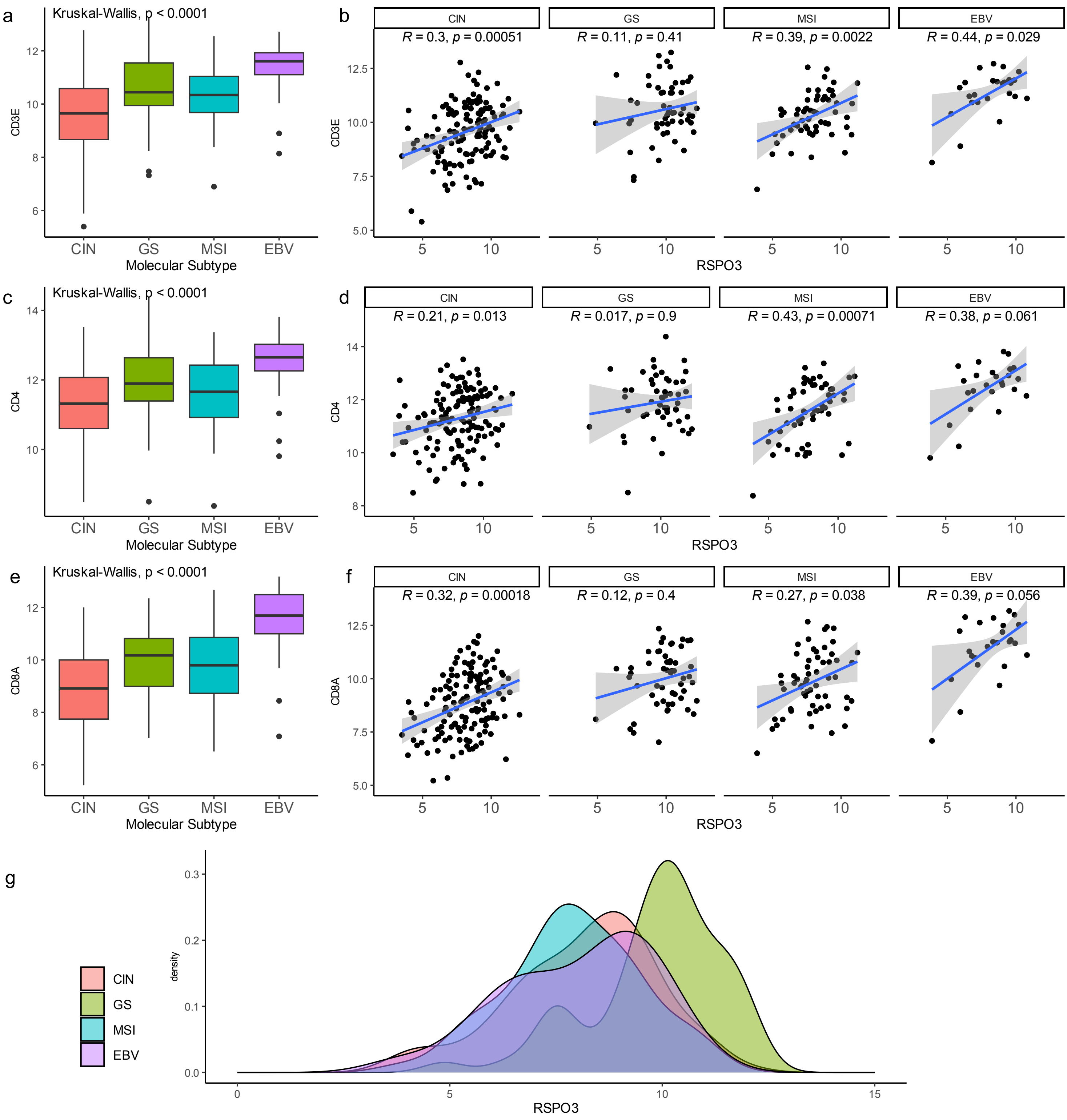

a, c, e) Abundance of a) CD3+ T-cells, c) CD4+ T-cells, and e) CD8+ T-cells in the four molecular subtypes of gastric adenocarcinoma in the TCGA cohort.

b, d, f) Scatter plots depicting the correlation between RSPO3 expression and abundance of b) CD3+ T-cells, d) CD4+ T-cells, and f) CD8+ T-cells in the four molecular subtypes of gastric adenocarcinoma in the TCGA cohort.

g) Expression of RSPO3 in the four molecular subtypes of gastric adenocarcinoma in the TCGA cohort.

CIN=Chromosomal Instability, GS=Genomically Stable, MSI=Microsatellite Instability, EBV=Epstein-Barr Virus Positive

Supplementary Table 1: Patient data

		GC			AEG		
		Rspo3 low	Rspo3 high	P value	Rspo3 low	Rspo3 high	P value
**Sex**	Male	74	74	0.41	15	14	0.21
	Female	48	37		11	4	
**Age**	<63 years	58	55	0.79	19	6	0.01
	>=63 years	64	56		7	12	
**Tumor stage**	T1	17	15	0.69	7	3	0.25
	T2	50	45		7	9	
	T3	37	40		11	4	
	T4	17	11		1	2	
**Nodal status**	N0	37	29	0.90	10	3	0.13
	N1	36	33		6	10	
	N2	25	25		4	1	
	N3	24	24		4	4	
**Metastatic status**	M0	87	80	1	22	13	0.45
	M1	35	31		4	5	
**Grading**	G1	0	1	0.38	0	0	0.74
	G2	27	30		7	6	
	G3	95	80		19	12	
**Lauren classification**	Intestinal	37	39	0.23	10	10	0.42
	Diffuse	73	55		12	7	
	Mixed	12	17		4	1	
**Ming classification**	Expansive	47	42	0.90	10	12	0.12
	Infiltrative	73	68		16	6	
**UICC classification**	Ia	15	12	0.97	6	2	0.32
	Ib	15	13		0	1	
	IIa	20	20		8	7	
	IIb	18	21		2	3	
	IIIa	11	8		2	0	
	IIIb	5	5		3	0	
	IIIc	3	1		1	0	
	IV	35	31		4	5	
**CPS**	Low	95	75	0.04	19	13	0.70
	high	15	26		4	4	

Supplementary Table 2: Absolute numbers of RSPO3 low/high tumors

		GC + AEG	GC	AEG
**RSPO3 status**	RSPO3 low	148	122	26
	RSPO3 high	129	111	18
